# Data-driven modeling of core gene regulatory network underlying leukemogenesis in IDH mutant AML

**DOI:** 10.1038/s41540-024-00366-0

**Published:** 2024-04-09

**Authors:** Ataur Katebi, Xiaowen Chen, Daniel Ramirez, Sheng Li, Mingyang Lu

**Affiliations:** 1 Department of Bioengineering, Northeastern University, Boston, MA USA; 2 Center for Theoretical Biological Physics, Northeastern University, Boston, MA USA; 3 Jackson Laboratory for Genomic Medicine, Farmington, CT USA; 4 Department of Computer Science & Engineering, University of Connecticut, Storrs, CT USA; 5 The Jackson Laboratory Cancer Center, Bar Harbor, ME USA

**Keywords:** Regulatory networks, Dynamic networks, Cancer

## Abstract

Acute myeloid leukemia (AML) is characterized by uncontrolled proliferation of poorly differentiated myeloid cells, with a heterogenous mutational landscape. Mutations in IDH1 and IDH2 are found in 20% of the AML cases. Although much effort has been made to identify genes associated with leukemogenesis, the regulatory mechanism of AML state transition is still not fully understood. To alleviate this issue, here we develop a new computational approach that integrates genomic data from diverse sources, including gene expression and ATAC-seq datasets, curated gene regulatory interaction databases, and mathematical modeling to establish models of context-specific core gene regulatory networks (GRNs) for a mechanistic understanding of tumorigenesis of AML with IDH mutations. The approach adopts a new optimization procedure to identify the top network according to its accuracy in capturing gene expression states and its flexibility to allow sufficient control of state transitions. From GRN modeling, we identify key regulators associated with the function of IDH mutations, such as DNA methyltransferase DNMT1, and network destabilizers, such as E2F1. The constructed core regulatory network and outcomes of in-silico network perturbations are supported by survival data from AML patients. We expect that the combined bioinformatics and systems-biology modeling approach will be generally applicable to elucidate the gene regulation of disease progression.

## Introduction

AML, the most common acute leukemia in adults, is characterized by uncontrolled proliferation of poorly differentiated and immature myeloid cells. Three classes of mutations have been observed in leukemic myeloid cells^1^. Class I mutations are followed by class II mutations, contributing to about 80% of the AML cases. Class I mutations lead to the activation of receptor tyrosine kinases FLT3, KIT, and RAS signaling pathway, inducing cellular proliferation. Subsequent class II fusion mutations RUNX1/ETO, CBFB/MYH11, and PML/RARA affect transcription factors (TFs) RUNX1, CBFB, and PML and compromise normal differentiation. Class III mutations are found in genes encoding epigenetic modifiers such as DNMT3A, IDH1, IDH2, TET2, ASXL1, and EZH2, and can cause leukemia with worse patient outcome^1^. Specifically, mutations in IDH1 and IDH2, two genes encoding the cytoplasmic and mitochondrial forms of isocitrate dehydrogenase, respectively, are found in about 20% of AML cases^2^. These mutations contribute to a hypermethylated state in AML^3^. Moreover, IDH mutations and TET2 mutations are mutually exclusive^3,4^ and IDH-mutant methylation and gene expression profiles are similar to those in TET2-mutant AML, suggesting a common pathogenic pathway^3^.

Although much effort has been made to elucidate the mutational landscape of AML and the linkage between these AML-associated mutations and disease severity, the gene regulatory mechanism of leukemogenesis is not yet fully understood. AML is a complex disease that arises from misregulation of gene regulatory network (GRN) driving normal cellular differentiation^5^. Therefore, mathematical modeling of the underlying GRN of AML and the effects of genetic perturbation can elucidate the gene regulation of the disease process and shed lights on new therapeutic strategies for AML. Some recent GRN modeling studies made efforts to elucidate AML gene regulation^6–12^. For example, Wooten et al. constructed a GRN of 106 nodes and 270 edges by composing interactions from different sources (e.g., SIGNOR) and performed Boolean modeling of the network to study drug response in class I FLT3 mutated AML^11^. Another recent Boolean network modeling study has refined a GRN model to recapitulate cellular state transitions during early hematopoiesis aging^13^. Despite the success of these modeling efforts, what is still missing is an approach that allows to systematically establish mechanistic models of GRN driving a specific subtype of AML. A promising solution to this question is to integrate top-down bioinformatics approach and bottom-up mathematical modeling for constructing GRNs of key transcription factors (TFs), referred as core GRNs^14^. A recently developed method, named NetAct^15^, has adopted this approach for modeling core GRNs driving cellular state transitions using gene expression data of multiple states and literature-based TF-target databases. Further generalization of this approach to integrate context-specific transcriptomics and epigenomics datasets and to enable GRN model selections based on network dynamics would allow to improve its capability for generating high-quality context-specific network models.

Here, we developed a new data-driven approach to inferring and modeling core GRN regulating leukemogenesis in IDH1/2 mutated AML by integrating top-down bioinformatics approach and bottom-up mathematical modeling. We first integrated data from diverse sources, including a microarray gene expression dataset, an ATAC-seq (Assay for Transposase-Accessible Chromatin using sequencing) data set for genome-wide chromatin accessibility, and literature-based databases containing TF to target gene relationship, to infer putative GRNs. For each GRN, we then applied a mathematical modeling method named *ra*ndom *ci*rcuit *pe*rturbation (RACIPE)^16–19^ to simulate the expression profiles of network genes for an ensemble of models with diverse kinetic parameters. The modeling approach has been streamlined to allow for a high-throughput application to many GRN topologies derived from the bioinformatics methods. We then identify the optimal GRN model where simulated gene expression data best match the experimental data, and meanwhile the GRN is sufficiently flexible to allow control of state transitions. From the established optimal GRN, we performed network perturbation modeling to identify key regulators associated with the mechanistic function of IDH mutations, such as DNMT1, and network destabilizers, such as E2F1, which are supported by patient survival data. Our modeling analysis further identifies the presence and coupling of key biological pathways, such as cell cycle, AMPK, and p53 pathways. In short, the combined bioinformatics and systems biology modeling approach has allowed to uncover key factors underlying leukemogenesis.

## Results

### An integrative network modeling framework

**Fig. 1:**
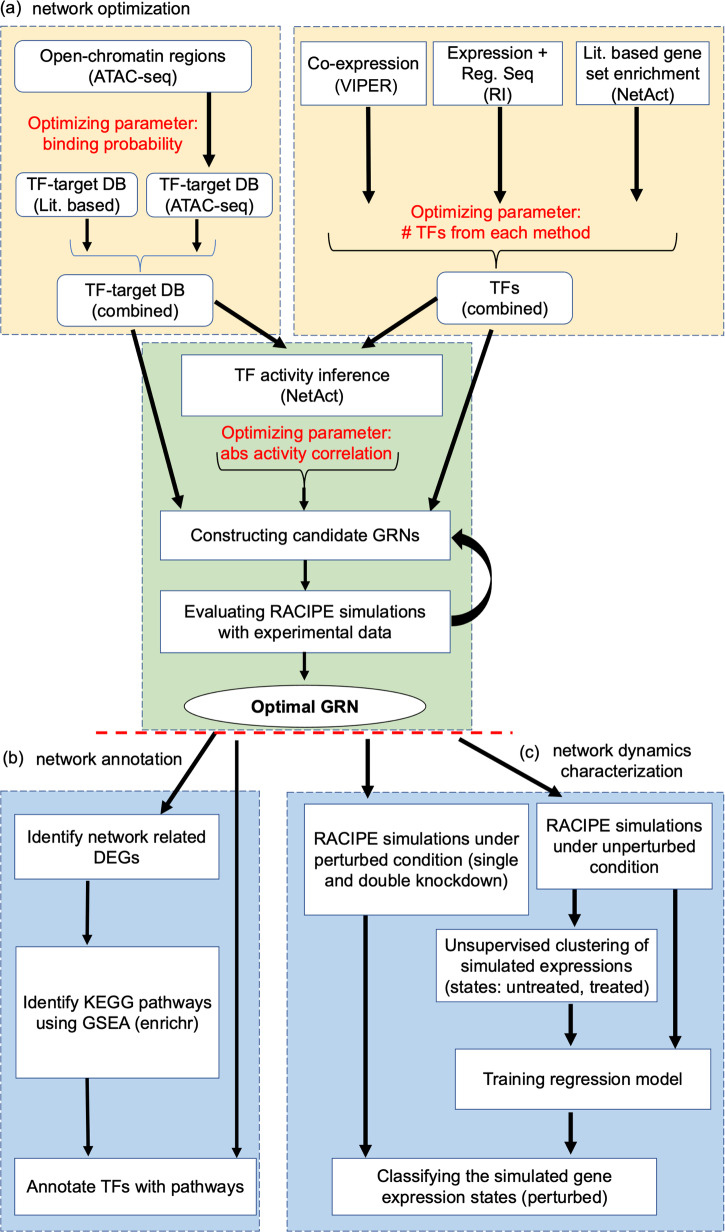
Illustration of the computational framework for gene regulatory network inference, optimization, and modeling. **a** Procedures for gene regulatory network (GRN) inference and optimization. The top left block shows the steps to construct TF-target databases (DB) using a literature-based TF-target DB and the TF-target relationships inferred from ATAC-seq data. The top right block shows the approach of TF inference using three distinct computational methods: VIPER, RI regression, and NetAct. The bottom block shows the steps to construct GRN candidates using the TF-target databases and TF activities. Many candidate GRNs are constructed by varying three adjustable hyperparameters, as highlighted in red color. Network optimization is then applied to identify the optimal GRN that best captures experimental gene expression states according to GRN simulations by RACIPE, while also maintaining flexibility to allow network state transitions. **b** Network annotation. Using the GRN-related differentially expressed genes (DEGs), we identify enriched KEGG biological pathways and the best representative pathways associated with each network TF. **c** Network dynamics characterization. In silico network perturbation analysis can be further performed to identify key regulators of the network driving state transitions.

We designed a new computational network modeling framework that integrates bioinformatics methods with mathematical modeling to infer context specific gene regulatory networks (GRNs). The framework consists of the following steps, as illustrated in Fig. 1 and described in detail in Methods. First, key TFs are identified by applying three distinct network construction methods, namely VIPER^20^, RI^21^, and NetAct^15^. Second, a context-specific TF-target database is constructed by combining curated TF-target databases and TF-target gene relationship derived from ATAC-seq data. Third, the activity of each key TF is inferred by NetAct using the expression of their corresponding target genes. Fourth, a GRN consisting of the combined TFs from three different methods is constructed, where a regulatory link between two TFs is determined by both the context-specific TF-target database and the correlation of the activities of the TFs. We sampled three network construction parameters, namely ATAC-seq TF-binding probability cutoff, number of TFs taken from each TF selection method, and correlation cutoff of TF activities (Fig. 1a), which generated 532 candidate GRNs. Subsequently, we applied the mathematical modeling method RACIPE^17^ to each GRN to evaluate how well the GRN steady states capture the TF activity profiles from both the normal controls and the AML patients and how flexibly the GRN drives transitions between normal and disease states, from which we identified an optimal GRN. Furthermore, we used enrichr^22^ to find the significantly enriched biological pathways in the differentially expressed genes and annotated the TFs with the most representative pathways (Fig. 1b). Finally, network simulations and gene perturbation analyses were performed on the optimal GRN to predict the key regulators, which can be potential therapeutic targets of AML (Fig. 1c).

### Inference and optimization of a core AML GRN

**Fig. 2:**
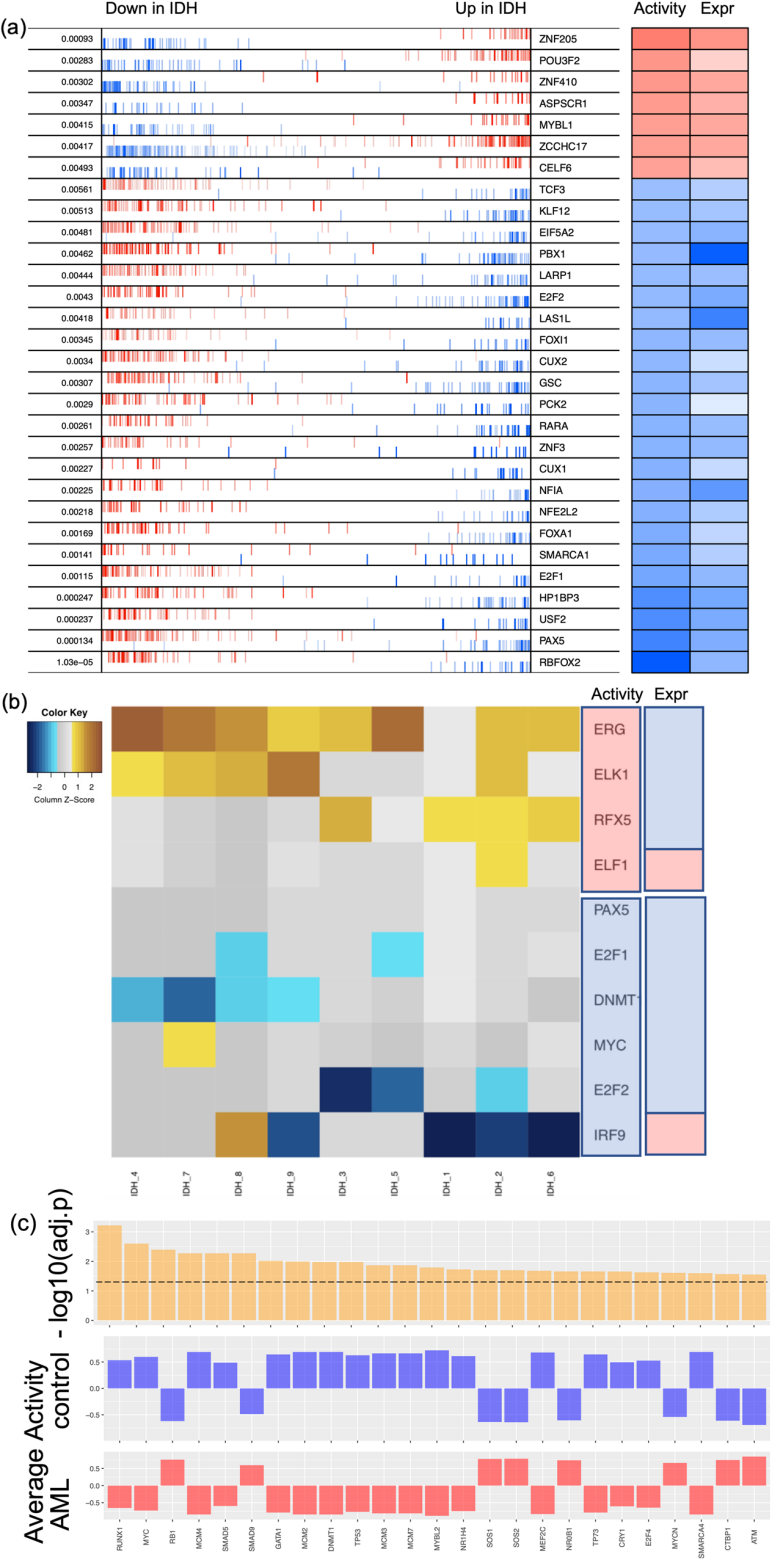
Identified transcription factors from three inference methods. **a** VIPER: Left side of the plot shows the distribution of the positively (red) and negatively (blue) correlated targets for each selected TF on the gene list ranked from the one most down-regulated to the one most upregulated in AML samples with *IDH* mutations compared with the samples of normal control. The two-column heatmap on the right side shows the inferred differential activity (first column labeled as Activity) and differential expression (second column labeled as Expression). **b** RI: The heatmap shows the AML sample-specific lasso model coefficients for each selected TF. In the annotation bars from the right side, the first column shows the activity of the TFs, and the second column shows the gene expression of the TFs (pink denotes upregulation, and blue denotes downregulation). **c** NetAct: 1st row shows −log10(adjusted *p* value) of the top 25 TFs ordered based on adjusted *p* values; 2nd row shows the average activities of normal control samples; 3rd row shows the average activities of IDH samples. A horizontal dotted line represents adjusted *p* value = 0.05.

In this study, we used a previously published microarray gene expression data from nine AML patients with IDH1/IDH2 mutation and without DNMT3A mutation and eleven normal controls from normal bone marrow CD34+ hematopoietic stem and progenitor cell (HSPC) specimens^23,24^. Using these data, we inferred key TFs by applying three distinct network construction methods. First, we obtained a ranked TF list by applying VIPER^20^, which assesses TF activity by combining transcriptional activation of its activated and repressed targets and its biological relevance by the targets overlapping with phenotype-specific programs (Fig. 2a). We obtained the second TF list by applying the regulator inference (RI)^21^, a lasso regression-based method, to the gene expression data and the TF motif binding sites from the ATAC-seq datasets for leukemia stem cells from seven AML patients^25^. This RI method assigns importance score to each TF (Fig. 2b). We then obtained the third TF list by applying NetAct^15^, which identifies the enriched TFs by performing gene set enrichment analysis (GSEA, with slight adjustments^15^) using a curated TF-target database on the differentially expressed genes (defined as those with the adjusted p-values below 0.05 by using *limma*^26^) between the normal controls and the AML patients with IDH mutations (Fig. 2c). These three methods (VIPER, RI, and NetAct) utilize different input datasets (Supplementary Table 1) and capture different aspects of the underlying regulatory mechanism (see Methods section “*Inference of transcription factors*”**)**.

From the inferred TFs by each method, we obtained many candidate GRNs of different sizes as follows. First, we constructed a combined TF-target gene-set database, which included literature-based TF-target gene sets and the TF-target gene relationships obtained from the ATAC-seq data. Next, we employed NetAct to calculate the activities of the selected TFs using the expression of their corresponding target genes, as defined by the combined TF-target database. Then, candidate GRNs were inferred according to the Spearman correlation between TF activities. The rationale behind using the TF activity, but not the expression, is that aberrant TF behavior in the disease state may not get manifested in the differential gene expression of the TF, rather in the coordinated activation of the target genes^27,28^. We obtained 532 candidate GRNs (examples in Supplementary Fig. 1) by varying the hyperparameters—namely, the number of TFs selected from each method (VIPER, RI, NetAct), the ATAC-seq TF-target gene binding probability, and the TF activity correlation cutoff (see Supplementary Table 2). Lastly, we systematically applied mathematical modeling to each candidate GRN for network optimization. Here, we applied RACIPE^17^ to each candidate GRN to generate an ensemble of 10,000 ordinary differentiation equation (ODE) models with randomly generated kinetic parameters (see Methods section “*Simulation of GRN using RACIPE”)*. Compared with the conventional modeling approaches where a set of kinetic parameters needs to be specified, RACIPE uses the topology of a GRN as the only input and identifies the network states from the gene expression clusters observed in the simulated gene expression profiles. Some previous studies have demonstrated that RACIPE can captures experimentally observed cellular states from an ensemble of randomly generated models^16,18,29–32^.

**Fig. 3:**
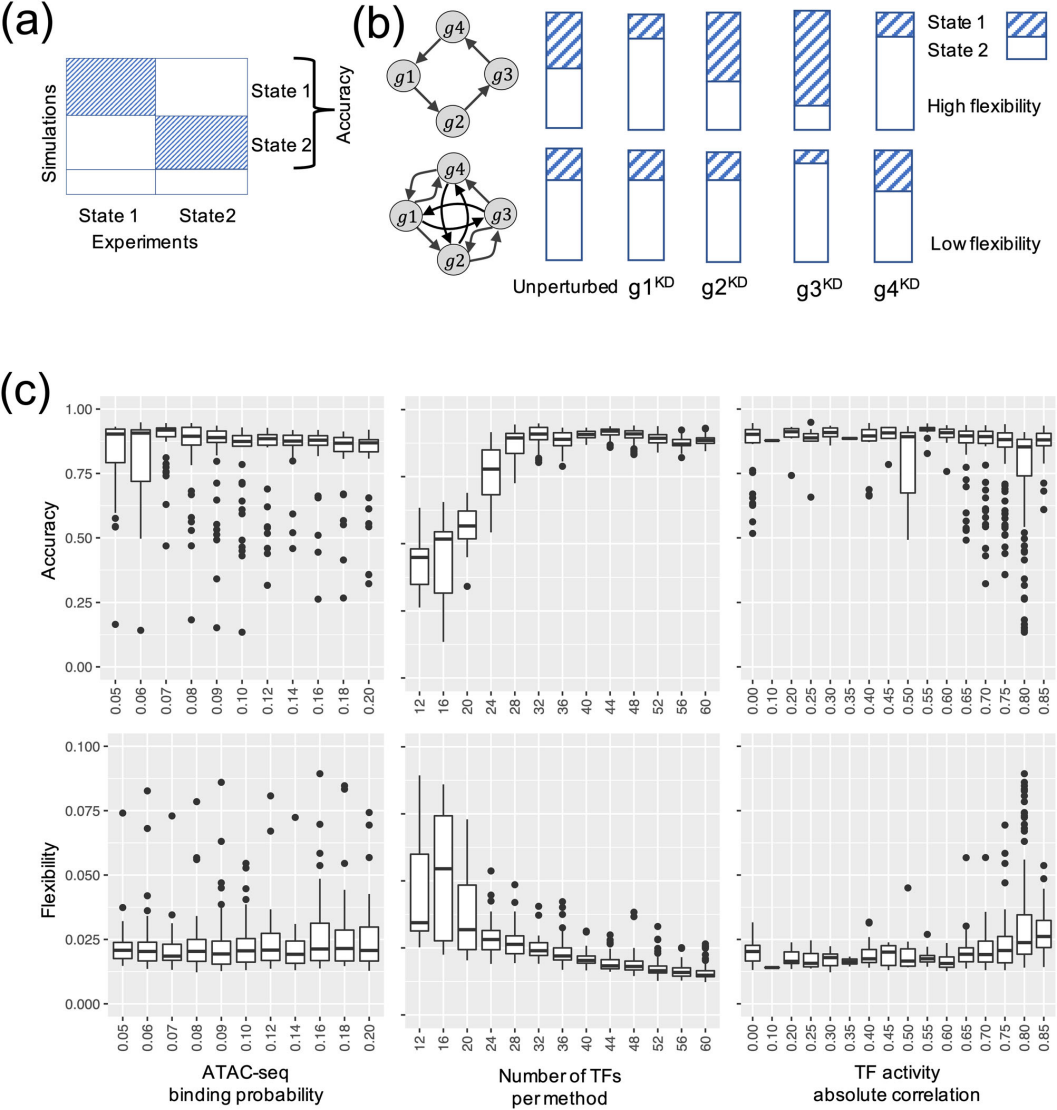
Network optimization by the accuracy and flexibility scores. **a** Schematic for the definition of accuracy. Accuracy is defined as the fraction of the RACIPE models that can be assigned to any of the two clusters (states 1 and 2) of gene expressions. **b** Schematic for the definition of flexibility. Flexibility is measured by the average deviation of the proportional of models in the two states between the perturbed and unperturbed conditions over all gene knockdown simulations. The circuit with larger average deviation (top) is more flexible than the other circuit (bottom). **c** Distribution of accuracy (top panel) and flexibility (bottom panel) of candidate GRNs with respect to the optimization hyperparameters: TF-binding probability (leftmost panels), number of TFs per method (middle panels), and the correlation cutoff (absolute value) of TF activity (rightmost panels). Box plots show median values at the center line, first and third quartiles within the box, and 1.5 times the interquartile range for whiskers, with remaining points defined as outliers.

**Fig. 4:**
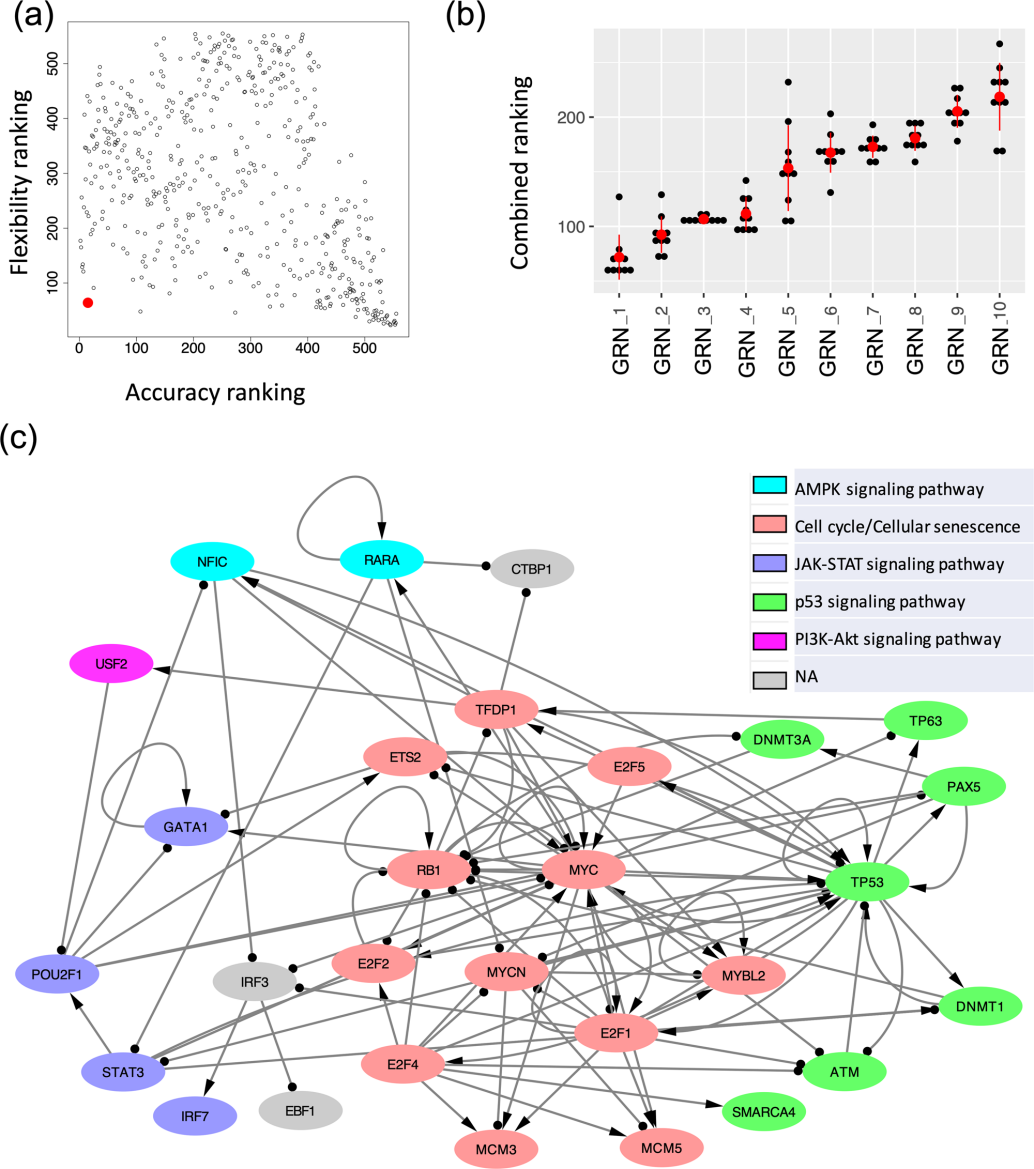
The optimal GRN of leukemogenesis in IDH mutant AML. **a** A scatter plot showing the accuracy ranking (x-axis) and flexibility ranking (y-axis) for a total of 532 GRN candidates. The optimal GRN is marked with the red enlarged dot. **b** Convergence of the top ranked networks. Distribution of the combined scores (sum of two rankings, one based on accuracy and the other based on flexibility) for the top ten GRNs obtained from the ten repeats of 10000 RACIPE simulations for each circuit. The red dot and the vertical bar are mean and standard deviation of the distribution for each circuit. A two-sided t-test shows that the scores for the top ranked GRN is significantly different from those of the other GRNs. **c** The diagram of the optimal AML GRN of enriched TFs, visualized using Cytoscape. Transcriptional activation is annotated by a line with arrowhead; transcriptional inhibition is annotated by a line with circle head. The colors of the gene nodes represent the most representative KEGG biological pathways. The coupling of biological pathways is illustrated in Supplementary Fig. 2.

Using the simulated gene expression profiles from the candidate GRNs, we then ranked each GRN with two metrics, namely accuracy and flexibility. Here, the accuracy of a GRN is calculated as the proportion of the RACIPE-simulated gene expression profiles that match the experimental TF activity profiles^32^ (Fig. 3a). The accuracy metric determines how well the simulation of a candidate GRN reconstructs the experimental data. We also defined flexibility^33^, which measures the average deviation of the proportional of models in the two states (i.e., normal and AML states) between the perturbed and unperturbed conditions over all gene knockdown simulations. A network with fewer connections will have higher flexibility than a dense network (Fig. 3b). See Methods section “*Accuracy and flexibility metrics*” for the calculation details. The distributions of accuracy and flexibility across the three network construction hyperparameters are shown in Fig. 3c. The optimal GRN is expected to exhibit high accuracy to capture the gene expression states and high flexibility to allow flexible control of state transitions^33^. Here, the accuracy metric captures the robustness of a GRN in creating and maintaining biological cellular states, while the flexibility metric characterizes how controllable the transitions between these states are. We expect functional GRNs to be sufficiently flexible because cellular state transitions can be controlled by cell signaling or gene perturbation. Therefore, we ordered the candidate GRNs based on the sum of the ranking indices of both accuracy and flexibility metrics. Figure 4a shows the scatter plot of accuracy ranking versus flexibility ranking, where the optimal network with the lowest index is highlighted in red. Additionally, the optimal GRN stays as the top network over repeated simulations and re-ranking and is significantly different from the second-best networks (*t* test, *p* value < 0.05, Fig. 4b), suggesting convergence of the network optimization. The optimal GRN consists of 29 TFs and 102 regulatory interactions, of which 53 are excitatory and 49 are inhibitory (Fig. 4c). In the optimal GRN, 28% of the interactions are derived from the ATAC-seq data (28 out of 102 interactions).

### Simulations of the core GRN agrees well with the experimental data

**Fig. 5:**
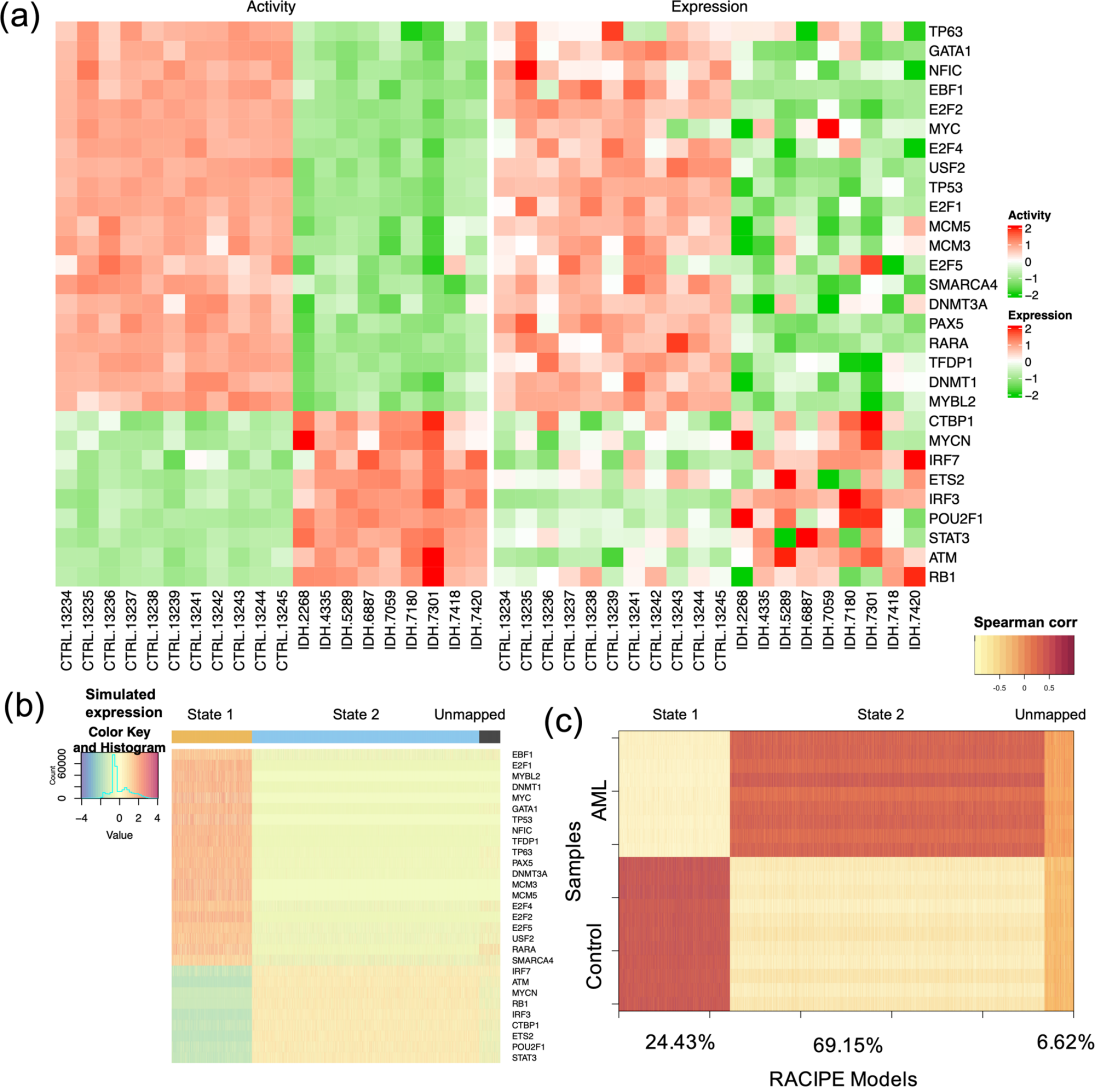
Simulation and characterization of the AML GRN. **a** Heatmaps showing the profiles of TF activities (left panel) and experimental gene expressions (right panel) for the TFs in the optimal core GRN. For row clustering, Euclidean distance and complete linkage method were applied. The columns show sample names for the control and the AML samples. **b** Heatmap of the RACIPE simulated gene expression profiles for the core GRN. Hierarchical clustering analyses were performed with the distance of one minus Spearman correlation and complete linkage. **c** Spearman correlations between the TF activities across samples (11 normal controls and 9 AML patients) along y-axis and the RACIPE simulated gene expressions along x-axis. The percentages (24.43%, 69.15%, and 6.62%) along the x-axis are the percent of the RACIPE models that are mapped to control group, treatment group, and neither of the two groups, respectively.

We used NetAct to calculate the activities of the 29 TFs in the optimal core GRN for the normal controls and the *IDH*-mutant AML patients. From the profiles of the activities and the expressions of the TFs in the GRN (Fig. 5a), it is evident that the TF activity profiles can distinguish the normal controls and the AML patients well. Furthermore, RACIPE simulation of the core GRN shows high agreement with the experimental data. Here, to perform the similarity analysis, we generated 10000 gene expression profiles from RACIPE simulations of this network and then mapped the models to the TF activity profiles of either the normal controls or the AML patients (See Methods section “*Accuracy and flexibility metrics*” for profile mapping details). There is a subset of the RACIPE models (Fig. 5b, cluster with black marker at the top-right) that could not be mapped to any of the two groups, normal controls and AML patients. The lower the proportion of these unmapped models, the better the GRN captures the gene expression states of normal and cancer conditions. The accuracy of the optimal GRN, measured as the percent of models that conform with the data, is 0.93, where the proportions of the models that match the normal and cancer conditions are 0.24 and 0.69, respectively (Fig. 5c).

### GRN modeling elucidates the drivers of leukemogenesis in IDH1/2 mutant AML

The core GRN associated with leukemogenesis in IDH1/2 mutant AML reveals the importance of DNMT1 as a key TF. Studies have shown that IDH1/2 mutations and TET2 mutations are mutually exclusive, resulting in an overlapping hypermethylation signature^3^. The oncometabolite 2-HG, produced by mutant IDH1/2, disrupts TET2 function and promotes oncogenesis^34^. Additionally, IDH1/2 mutations activate HDAC1/2, inhibiting the formation of the DNMT1 and TET2 complex, leading to the degradation of DNMT1 and TET2^35^. This impairment of the DNMT1 and TET2 complex formation contributes to abnormal DNA methylation in IDH-mutated AML. Moreover, the core GRN involves crucial cell cycle and DNA-damage-repair genes, such as RB1, E2F1/2, TP53, and MYC, and several stem cell pluripotency factors GATA1^36^, POU2F1, and MYCN^37^. The overexpression of these genes suggests that the AML cells attain stem cell like phenotype with a much-restricted cell cycle, which may induce drug resistance to these AML cells^38,39^. These TFs can also facilitate the coupling of multiple pathways to carry out the required complex biological functions.

### GRN modeling identifies the presence and coupling of key biological pathways

Furthermore, we identified six key KEGG pathways^40^ involving the TFs in the core GRN by performing GSEA using the TFs and their target genes (details in Methods section “*Pathway annotation*” and Supplementary Data 1 and 2). These enriched pathways include two regulatory pathways (cell cycle and cellular senescence) and four signaling pathways (AMPK, JAK-STAT, p53, and PI3K-AKT). Using Fisher’s exact test between the genes in a pathway and a TF’s regulon (defined as a gene set containing the TF and its target genes), we computed the significance of overlapping between them and annotated each TF in the optimal network with the most significant pathway (Fig. 4c). The coupling between these pathways is shown in Supplementary Fig. 2. JAK/STAT is the central communication node in cell function that is involved in cellular progression and differentiation together with hematopoiesis among other functions^41^. In a recent study, Habbel et al. found that JAK/STAT signaling pathway is activated because of the inflammation in the AML cells^42^. Also, AML enables the myeloid cells to proceed uncontrolled and limitless number of cell cycles^43^. Cellular senescence promotes the evasion of tumor cells from immunosurveillance^44^. The coupling of JAK-STAT signaling pathway and cell cycle suggests increased cell-cell communication and expedited cell growth, which is shown in recent in vitro experiments^42^. On the other hand, the activation of p53 signaling pathway coupled with cellular senescence can be attributed to the DNA damage^45^ and subsequent cell cycle arrest^46^ in leukemogenesis. PI3K-AKT signaling pathway plays a role in both cell proliferation and cell cycle arrest in AML^47^. AMPK exhibits a dual role in AML, as it acts as a tumor suppressor before the disease onset but can promote disease progression after its onset in association with other key pathways^48^. Together, the findings suggest that the coupled gene regulation of these signaling pathways contributes to tumorigenesis in AML.

### Perturbation analysis reveals significant TFs in the core GRN

**Fig. 6:**
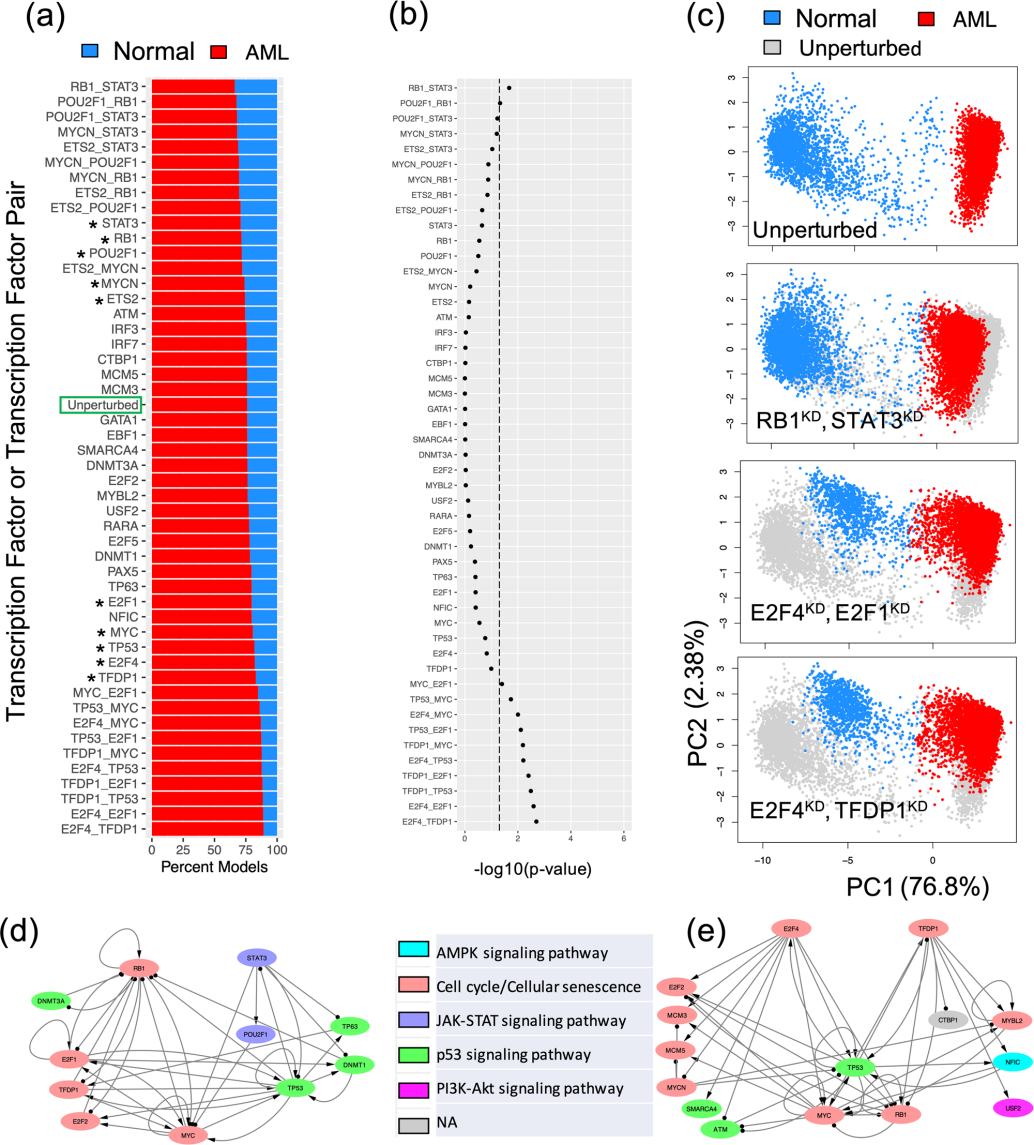
In silico perturbation analysis of the AML GRN. **a** Proportion of models (belonging to the two experimental groups -- normal controls and AML patients) from single and double knockdown (KD) simulations. Perturbations are arranged in descending order based on model proportions in the normal group. The top five and bottom five genes (marked by stars) from single KD simulations were used for double KD simulations. **b** Significance of changes in gene expression states upon GRN perturbations by a chi-squared test. X-axis represents −log10(*p* value). Dotted line indicates *p* value = 0.05. **c** Examples of changes in gene expression profiles upon GRN perturbations. The first row shows the scatter plot of the simulated gene expression profiles of the GRN under the unperturbed condition projected on the first two principal components of the data. The second to fourth rows show the scatter plots of the simulated gene expression profiles of the GRN under various perturbed conditions in the same PCA space. For the last three rows, perturbed expressions are shown (blue: normal, red: AML) on top of the unperturbed expressions (gray). Results for the top single KD perturbations are shown in Supplementary Fig. 4. **d** A subnetwork containing RB1, STAT3, and their target transcription factors. A double KD of RB1 and STAT3 causes the largest decrease of the models in the AML state. **e** A subnetwork containing E2F4, TFDP1, and their target transcription factors. A double KD of E2F4 and TFDP1 causes the largest increase of the models in the AML state.

With the established core GRN, simulations of gene perturbations can be performed to identify crucial TFs or TF pairs destabilizing the network states^18,49,50^. Here, we simulated the GRN with either single or double gene knockdown (KD), and, for each case, we evaluated the proportion of models belonging to the normal and the AML states of the GRN (Methods section “*Modeling GRN perturbations*”). When the proportion of models in the AML state increases, the gene(s) undergoing KD would be regarded as destabilizer(s) of the AML state. From single KD perturbations, the top five destabilizers of the AML state are TFDP1, E2F4, TP53, MYC, and E2F1; in contrast, the top five destabilizers of the normal state are STAT3, RB1, POU2F1, ETS2, and MYCN, as shown in Supplementary Fig. 3. These top 10 destabilizers are associated with three key biological pathways: JAK-STAT signaling (STAT3, POU2F1), Cell cycle (TFDP1, E2F4, MYC, E2F1, RB1, ETS2, MYCN), and p53 signaling (TP53). Activation of JAK-STAT signaling and cell cycle indicates increased cell cycle communication and cell growth^42^, requiring activation of p53 signaling for repairment of increased DNA damage^45^. These top destabilizers from both directions were then used for double KD simulations. As expected, the double KDs have higher impact to the network states than the single KDs (Fig. 6a). Among all of the single and double KD simulations, 10 double KD perturbations were found to significantly expand the model proportions of the AML state (by a Chi-squared test, lower part of Fig. 6b).

Furthermore, we examined in detail how the network states change for the top three double KD perturbations (i.e., RB1-STAT3; E2F4-E2F1; E2F4-TFDP1) (Fig. 6c). First, we performed principal component analysis of the RACIPE-simulated gene expression profiles for the unperturbed condition and projected those profiles onto the first two principal components (PCs) (top panel in Fig. 6c). Next, the KD simulated gene expression profiles were projected onto the same PCs, as shown in the bottom three panels in Fig. 6c and Supplementary Fig. 4. Noticeably, the double KD of the TF pair RB1-STAT3 shifts the gene expressions of the AML models towards those of the normal models. On the other hand, the other two double KD perturbations, E2F4-E2F1 and E2F4-TFDP1, shift the gene expressions of the normal models towards those of the AML state. Hence, the perturbation analysis of the optimal GRN reveals the significant TFs and TF pairs that can shift the cell populations from AML state to normal state and vice versa. Such information can be important in designing effective therapeutic strategies.

To further examine the synergistic effects of the TF pairs in the double KD perturbations, we checked the two subnetworks consisting of the targets of TF pair RB1-STAT3 and TF pair E2F4-TFDP1, as shown in Figs. 6de. Here, the double KD of RB1-STAT3 has the largest impact to destabilize the normal state, while the double KD of E2F4-TFDP1 has the largest impact to destabilize the AML state. The E2F4-TFDP1 KD causes larger impacts to GRN states possibly because both TFs are on the largest pathway of the GRN (i.e., cell cycle) and have a higher number of overlapping target nodes, MYC, RB1, and TP53, in the GRN (Fig. 6e), whereas only one overlapping target node MYC for RB1 and STAT3 (Fig. 6d).

### Survival analysis suggests therapeutic strategies

**Fig. 7:**
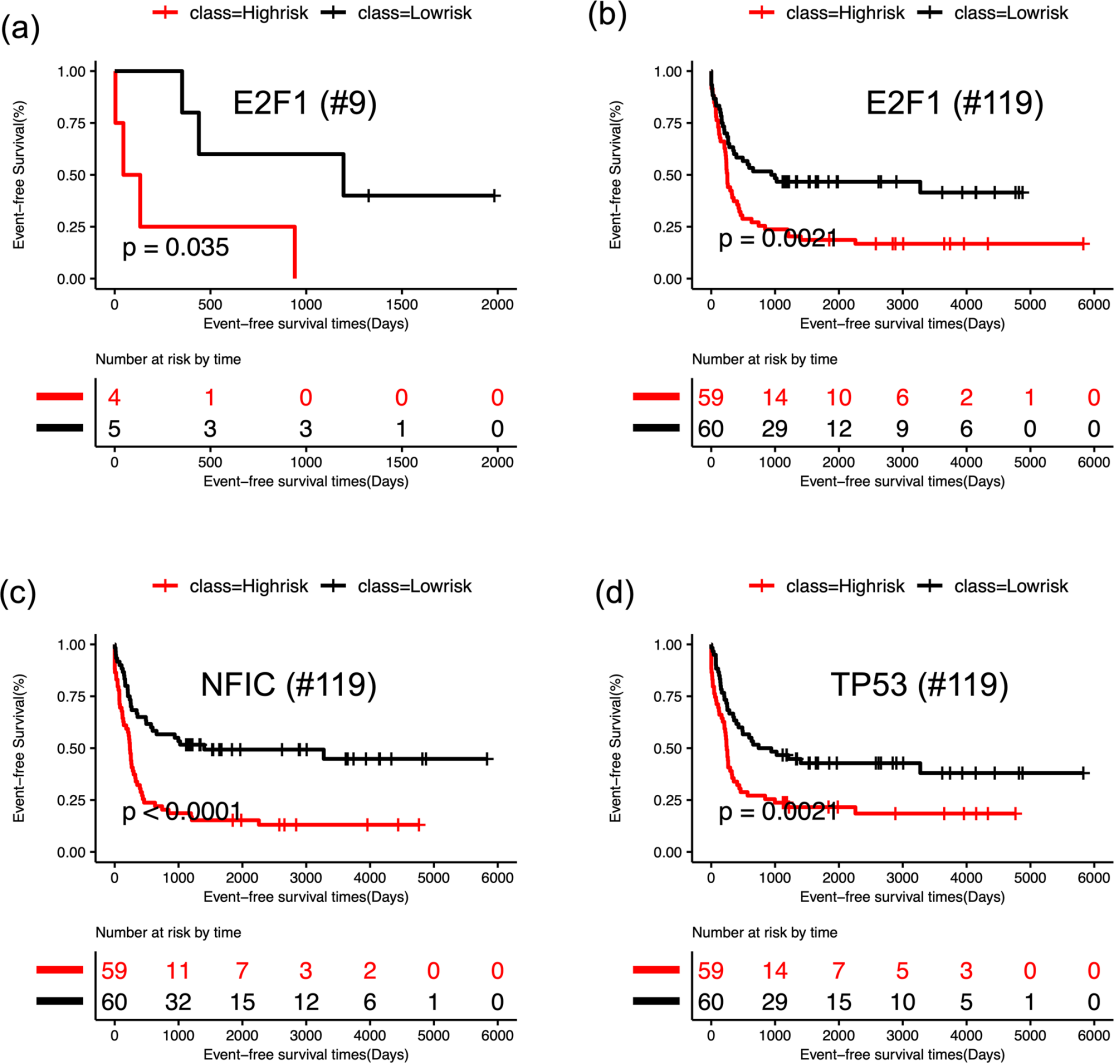
Survival analysis based on the gene expression of the core GRN. For each TF, log-rank test was performed to group the patients as high (red) and low risk (black) based on median of risk scores and then Kaplan-Meier analysis was performed for survival analysis. **a** Kaplan-Meier curves for event free survival for E2F1 using nine IDH AML patients. **b** Kaplan-Meier curves of event free survival for E2F1 using 119 AML patients. **c** Kaplan-Meier curves for event free survival for NFIC using 119 AML patients. **d** Kaplan-Meier curves of event free survival for TP53 using 119 AML patients. See Supplementary Fig. 5 for the survival analyses for additional TFs using all 119 AML patients.

To investigate the relationship of the 29 TFs in the GRN with the prognosis of AML patients, we performed Kaplan-Meier survival analysis and log-rank test on patients’ clinical data. We performed the survival analysis for two scenarios: in one case, we used only nine IDH mutant AML patients and, in the other case, we used all 119 AML patients. In each case, we calculated the risk score for each patient using the expression profiles of each individual TF and its target genes. We divided the AML patients into two groups (high-risk and low risk) based on their risk scores. For the key TFs, such as E2F1, NFIC, and TP53, a significant difference in event-free survival was observed between high- and low-risk groups (Fig. 7 and Supplementary Fig. 5). Additionally, these TFs were also found to be among the most impactful genes in the KD simulations (Fig. 6a–c and Supplementary Fig. 3). These results suggest that the identified TFs could act as prognostic factors of leukemia. Our observations are also supported by existing literature on AML studies. DNA methylation of E2F1 has been associated with clinical outcomes in distinct subtypes of AML^51^, although E2F1 has been proposed to be both oncogene and tumor suppressors in cancer^52^. In another recent study, Dutta et al. analyzed the TP53 mutation profiles of AML patients and found that AML patients with TP53 mutations showed worse prognosis than patients with wild type TP53^53^. GATA1, another prognostic factor found in our analysis, has been found to be epigenetically deregulated in AML^54^. This analysis further supports that the constructed core GRN included important TFs that are not only significant for IDH1/2 mutant AML leukemogenesis, but also predictive for the survival of other types of AML patients.

## Discussion

With the advent of high-throughput sequencing technology, large datasets of transcriptomic, proteomic, and genomic profiles of cancer patients, together with literature-curated gene regulatory interactions, have been available. Identifying the differentially expressed genes for cancer subtypes and the related enriched pathways does not clearly inform us the underlying gene regulatory mechanism of molecular state change in tumorigenesis. Despite the availability of plethora of molecular profiles of tumor samples, there is still a lack of suitable methodologies to extract important information from the diverse tumor datasets for a mechanistic understanding of tumorigenesis. Several top-down bioinformatics methods utilized high-throughput gene expression data to study dysregulation of gene expression in cancer^55,56^ and link the upstream signaling pathway to downstream transcription program^57^. Some other methods infer network of transcription factor and target genes by using multi-omics data^58–61^. Although the regulatory maps inferred by these methods give a global view of gene regulation, the generated networks usually do not correspond to a functional dynamical system to elucidate the gene regulation of the state transition between normal and cancer cells^14^. To address this issue, there is a need to develop approaches that allow to establish systems-biology gene network models for predicting gene expression dynamics directly from diverse cancer genomics data sets.

Here, we introduced a generally applicable computational framework by extending our recently published method, NetAct^15^ for modeling GRNs driving cellular state transitions during disease development by using a combined top-down bioinformatics and bottom-up mathematical modeling approach. The top-down approach was applied to generate a collection of putative GRNs by integrating genomics data from diverse sources. Subsequently, the bottom-up mathematical modeling approach was applied to identify the optimal GRN that reproduces experimental gene expression data. Compared to NetAct, the method presented here offers two key enhancements. First, it integrates ATAC-seq data and literature-based curated TF-to-target gene relationships, whereas NetAct solely relies on the curated database. Second, the current method employs mathematical modeling to identify the optimal gene regulatory network (GRN) among many candidate GRNs. Empowered by these improvements, the current method enables us to find the optimal GRN that elucidates the gene regulatory mechanism of leukemogenesis in AML and unravels the coupling of relevant biological pathways. In particular, the method successfully captures a key regulator DNMT1, a known factor associated with IDH1/2 functions^35^. The optimal GRN also identifies key genes involved in cell cycle regulation and DNA damage repair, such as RB1, E2F1/2, TP53, and MYC, along with stem cell pluripotency factors STAT3, POU2F1, and MYCN. Overexpression of these genes suggests that AML cells acquire a stem cell-like phenotype with a restricted cell cycle, potentially leading to drug resistance. In addition, the single and double knockdown simulations of the GRN identified E2F1 as one of the top TFs whose knockdown significantly increased the cancer state, which is supported by the survival analysis of the AML patients.

While our approach has yielded promising results, several limitations warrant investigation for future advancements. We currently applied our approach to study AML tumorigenesis whereas the dataset captures mainly two cellular states. It would be interesting to apply such an approach to systems where one or multiple intermediate states are captured in the data and systems with complex structures of cellular state transitions, such as those during cell fate reprogramming^62^. Additionally, the integration of multiomics datasets, such as microarray gene expression data and ATAC-seq chromatin accessibility data obtained from separate experiments, may benefit from the generation of multimodal datasets, where both datasets are obtained from the same cells. Such integration would enhance the context-specificity of inferred GRNs. Furthermore, other valuable data types, like Hi-C data, could offer regulatory information not currently accounted for in our method. Another consideration pertains to the time-consuming nature of simulating all potential GRNs to identify the optimal network, especially when dealing with a substantial number of inferred GRNs. This can be mitigated by parallelizing the simulations of potential GRNs, which can significantly reduce the computation time. Implementing this parallelization would enhance the efficiency and scalability of our approach, making it more practical for larger datasets and complex analyses.

Despite these limitations, our current approach marks a valuable steppingstone in exploring gene regulatory networks as systems biology network models. Addressing these considerations in future research will undoubtedly improve the method’s capabilities, enabling it to deliver even more comprehensive and accurate insights into the regulatory mechanisms of cellular state transitions.

## Methods

### Preprocessing gene expression and ATAC-seq data

We used a previously published microarray gene expression data for the primary AML patients (*n* = 119) and a control group from normal bone marrow CD34+ hematopoietic stem and progenitor cell (HSPC) specimens (*n* = 11), which was profiled using Affymetrix Human Genome U133 Plus 2.0 GeneChips (Gene Expression Omnibus (GEO) accession number GSE6891)^23,24^. The raw data were reprocessed using the HGU133plus2.0 BrainArray annotation version 17.0.0. Gene expression levels were transformed to log2 values. Network modeling analyses were applied to the data for *IDH-*mutant AML patients (*n* = 9, IDH1/IDH2 mutation and without DNMT3A mutation) and the normal controls to identify context-specific TFs.

We utilized ATAC-seq data to identify open chromatin regions within the promoter region, enabling the identification of context-specific TF-target relationships. The ATAC-seq datasets for leukemia stem cells from seven AML patients were obtained (GEO with accession number GSE74912)^25^. Sequencing data were pre-processed by the interactive-ATAC (I-ATAC) pipeline^63^. Briefly, we used *Trimmomatic*^64^ to identify and trim adapter sequences and low quality nucleotide sequences from the raw ATAC-seq read. Trimmed reads of each sample were mapped to the human reference genome GRNh37/hg19 by *BWA*^65^. *Picard*^66^ was used to filter PCR duplicated reads and calculate inset size. Next, I-ATAC adjusted sequencing as described by pipeline and the outcome was converted into the BED format to identify genomic regions enriched in the putative open chromatin sites (peaks) by *MACS*^67^. Finally, the ATAC peaks presented in all the seven AML patient datasets were used for TF binding site prediction.

### Inference of transcription factors

A list of TFs was obtained by applying each of the three previously published methods: Virtual Inference of protein-activity by Enriched Regulon analysis (VIPER)^28^, Regulatory Inference (RI)^21^, and NetAct^15^. Different datasets used by these methods are listed in Supplementary Table 1.

#### Preprocessing Rcistarget data for VIPER and RI methods

The cis-binding motifs for human transcription factors were collected from Rcistarget v1.3, which contains 982 transcription factors (TFs) and 1872 motifs. Position weight matrices were converted to the MEME motif format^68^ and the FIMO tool from the MEME package was used to search for binding sites at the open chromatin peaks within 2 kb upstream and downstream of the transcription start sites (*p* value < 0.0005). We used the default parameter of FIMO except for the max-stored-scores and motif-pseudo-options which we set to 100,000,000 and 1 × 10^−8^, respectively.

#### VIPER

First, we used the function *aracne2regulon* from ARACNe algorithm^69^ to generate context-specific regulatory network based on gene expression of AML patients with IDH mutation and CD34^+^ controls. Then, the *msviper* function in viper R package is used to generate normalized enrichment score (NES) and p-value, which identified 230 key IDH-specific TFs with FDR-adjusted *p* value less than 0.05.

#### RI (sample-by-sample lasso regression models)

We used sample-by-sample lasso regression models from the RI method^21^ with inputs gene expression profile and regulatory sequence information to infer sample-specific TF activities and IDH-specific key regulators. Here, we used linear regression to model log gene expression changes in AML patients with IDH mutation versus CD34^+^ controls by TF binding site counts in the gene promoter as variate. Quantification of binding site counts from ATAC-seq data can be found in the Methods section “*Preprocessing gene expression and ATAC-seq data*”. Lasso regression was performed using the glmnet function in the R package^70^. The regularization parameter was determined using tenfold cross-validation for each sample. The coefficient of each TF estimates the importance of the TF in the sample. We performed feature dependency analysis using RI method to obtain 938 key IDH-specific TFs.

#### NetAct

We employed our newly developed method NetAct^15^ for TF selection from the gene expression data from 11 normal controls and 9 AML patients with IDH mutation^23,24^ and a TF-target gene database. First, a two-way comparison (normal control and IDH mutation condition) was performed for differential gene expression (DE) analysis using limma^26^ (using the function *DEG_Analysis_Micro* provided in NetAct). This generated a ranked gene list quantified by adjusted *p* value. Then, the enriched TFs were identified by performing gene set enrichment analysis (NetAct function *TF_Selection*, with slight modification on GSEA, number of permutations = 1000) using our curated TF-target gene database. The curated TF-target gene database was compiled from different sources as listed in Supplementary Table 1. For GSEA, we considered 312 TFs with eight or more targets in the NetAct TF-target gene database and obtained the TFs ranked by adjusted p-value.

Each of these methods (VIPER, RI, and NetAct) was applied independently to obtain a ranked list of TFs. We then combined TFs from the three lists to construct candidate gene regulatory networks (GRNs) (see Methods section “*GRN optimization*”).

### Integration of ATAC-seq data

We constructed TF-target databases by combining curated TF-target gene database with TF-target gene relationships obtained from the ATAC-seq dataset at different TF-target binding probability thresholds, with the aim of finding a balanced mix of curated and ATAC-seq targets. At each TF-target gene binding probability threshold, we selected the targets for each TF from the ATAC-seq data according to the following criteria:

1 $${n}_{{target}}=\left\{\begin{array}{c}{n}_{{genes}},{n}_{{genes}} \,<\, {N}_{{TSH}}\\ {N}_{{TSH}}+\left({n}_{{genes}}-{N}_{{TSH}}\right)* p,{n}_{{genes}} \,>\, {N}_{{TSH}}\end{array},\right.$$

where ${n}_{{genes}}$ represents the number of probable target genes above the TF binding probability threshold, ${N}_{{TSH}}$ represents the threshold for the number of probable target genes below which all are selected as targets (set at 50), and $p$ represents the percent of genes used to select the top target genes from the probable target genes (${n}_{{genes}}$) (set at 0.01 to select the top 1% target genes). Then, the inferred TF-target gene relationships for a specific TF-target gene binding probability threshold were merged with the curated TF-target gene database. We retained the TFs with at least eight targets in the merged TF-target gene database. Eleven TF-target gene binding probability thresholds were chosen: 0.05, 0.06, 0.07, 0.08, 0.09, 0.10, 0.12, 0.14, 0.16, 0.18, and 0.20 (Supplementary Table 2).

### GRN optimization

To construct candidate GRNs, we first inferred a list of core TFs as follows. First, we selected a specific number of TFs from each of the three bioinformatics methods (NetAct, VIPER, and RI). Then, we combined the TFs selected from each method at each ATAC-seq TF-binding probability cutoff. See Supplementary Table 2 for the choice of the hyperparameters number of TFs per method and ATAC-seq TF-binding probability cutoff. Here, we chose to include the same number of TFs from each method to balance the usage of different approaches. However, varying number of TFs could also be performed to sample more different GRNs. ATAC-seq TF-binding probability cutoff has also some impacts in this step, as the merged TF-target gene database was utilized for selecting TFs with at least eight target genes. The combined set of core TFs selected at each ATAC-seq probability cutoff and TF count per method, were then used for putative GRN construction as described below.

From a set of core TFs, we constructed an initial network set by connecting any TF pair from the combined TF-target gene database. Each regulatory interaction contains a regulator TF and target TF, and the interaction type could be either excitatory or inhibitory, determined by the sign of the Spearman correlation between the activities of the regulator and target TF pair. Only those interactions are retained whose absolute correlations are above a given threshold value. If the obtained network consists of multiple disconnected subnetworks, we retained the largest subnetwork containing more than 80% of the TFs from the obtained network. If the largest subnetwork is smaller than 80% of the obtained network, we discarded the network for optimization later. We repeated the above process for 20 TF activity Spearman correlation cutoff starting from 0.0 to 0.95 with a 0.05 stepwise increment. In this way, we retained 532 candidate GRNs with 15 or more TFs per network.

We obtained the optimal network among the 532 candidate GRNs according to the combined accuracy and flexibility ranking (as defined below in Methods section “*Accuracy and flexibility metrics*”). For each candidate GRN, 10,000 RACIPE models were first generated (see Methods section “*Simulation of GRN using RACIPE*”) to compute the accuracy and flexibility. The candidate GRNs were then ordered by the accuracy and flexibility (both from high to low), respectively. The combined index of a GRN was defined by the sum of the ordering indices of the accuracy and flexibility. Thus, the GRN with the smallest combined index was selected as the optimal GRN.

### Simulation of GRN using RACIPE

We applied a mathematical modeling method, Random Circuit Perturbation (RACIPE)^16^, to model the GRNs of transcriptional regulation (R package sRACIPE^17^). In RACIPE, for a gene $Y$ regulated by multiple regulators ${X}_{i}$ ($i=1,2\ldots$) transcriptionally, the dynamics of $Y$’s expression is given by an ordinary differential equation (ODE)

2 $${dY}/{dt}=\frac{{G}_{Y}}{\prod _{i}{{\lambda }^{+}}_{{X}_{i}Y}}{\prod}_{i}H^s({X}_{i},{X}_{i}{Y}_{0},{n}_{{X}_{i}T},{\lambda }_{{X}_{i}T})-{k}_{Y}Y,$$

where $Y$ and ${X}_{i}$ are the gene expression levels of genes $Y$ and ${X}_{i}$, respectively, ${G}_{Y}$ is the maximum production rate of gene *Y*, and ${k}_{Y}$ is the degradation rate of gene $Y$. $Hs$ is the shifted hill function for the $X$ to $Y$ regulation, with the expression,

3 $$Hs\left(X,{{XY}}_{0},{n}_{{XY}},{\lambda }_{{XY}}\right)={\lambda }_{{XY}}+{\left(1-{\lambda }_{{XY}}\right)}/({1+{\left(\frac{X}{{{XY}}_{0}}\right)}^{{n}_{{XY}}}}).$$

Here ${{XY}}_{0}$, ${n}_{{XY}}$ and ${\lambda }_{{XY}}$ are the threshold level, the Hill coefficient, and the maximum fold change for $X$ to $Y$ regulation. For an excitatory interaction, ${\lambda }_{{XY}}$ is denoted as ${{\lambda }^{+}}_{{XY}}$ (${{\lambda }^{+}}_{{XY}}\, >\, 1$), and $Hs$ takes the range of (1, ${{\lambda }^{+}}_{{XY}}$). For an inhibitory interaction, ${\lambda }_{{XY}}$ is denoted as ${{\lambda }^{-}}_{{XY}}$ (${{0 \,<\, \lambda }^{-}}_{{XY}} \,<\, 1$), and $Hs$ takes the range of (${{\lambda }^{-}}_{{XY}},1$). The term $\prod _{i}{{\lambda }^{+}}_{{X}_{i}Y}$ in Eq. 2 takes the product over all excitatory interactions of gene $Y$; the term functions as a scaling factor to ensure ${G}_{Y}$ has the meaning of the maximum production rate. RACIPE generates an ensemble of models with kinetic parameters randomly sampled from uniform distributions, i.e., ${G}_{Y}$ from (1, 100), ${k}_{Y}$ from (0.1, 1), ${n}_{{XY}}$ as integers from (1, 6), and ${{\lambda }^{+}}_{{XY}}$ from (1, 100). ${{\lambda }^{-}}_{{XY}}$ is first sampled from a uniform distribution of (1, 100) and then taken the inverse. ${{XY}}_{0}$ is selected from (0.02$M$, 1.98 M), where M is the median Hill threshold estimated by the half-functional rule^16^. For each ODE model, RACIPE simulates the gene expression dynamics of the whole network (Eq. 2 as an example for a target gene *Y*). The initial condition of the simulation is randomly selected from a logarithmic distribution for each gene $Y$ from a maximum of $\frac{{{\rm{G}}}_{Y}}{{k}_{Y}}$, and a minimum of $\frac{{{\rm{G}}}_{Y}}{{k}_{Y}}\left(\frac{\prod _{i}{{\lambda }^{-}}_{{X}_{i}Y}}{\prod _{i}{{\lambda }^{+}}_{{X}_{i}Y}}\right)$. Finally, we obtained the steady-state gene expression profile from each ODE simulation. A typical RACIPE analysis comprises of sampling and simulations of 10,000 random models, followed by the data analysis of simulated gene expression profiles. For the knockdown simulations, additional ODE simulation was performed for each RACIPE model, where selected gene(s) are expressed minimally. Here, we lowered the production rate of each knockdown gene by 95% and obtained the steady-state gene expression profile from each ODE simulation.

### Accuracy and flexibility metrics

Two metrics, accuracy and flexibility, were used to rank the candidate GRNs in the network optimization process. Accuracy captures the context specificity of a GRN by matching the RACIPE simulated gene expression with the experimental gene expression data, whereas flexibility captures the plasticity of the network by contrasting the RACIPE simulations under the unperturbed and perturbed conditions.

Accuracy of a candidate GRN was measured by the fraction of the RACIPE models (under the unperturbed condition) that can be assigned to any of the two experimental gene expression states (normal controls and AML patients). To assign a RACIPE model to an experimental state, first we calculated the Euclidean distance between the simulated gene expression profile of the RACIPE model and the nearest TF activity profile of a sample from the experimental state. Second, we generated 1000 random gene expression profiles by shuffling gene names and calculated each profile’s distance to the nearest TF activity profile of a sample from the experimental state. Using the distances from the random profiles as the null distribution, we calculated the p-value for each RACIPE model to be in that experimental state. Finally, we mapped each RACIPE model to the experimental state with the smallest *p* value. If the *p* values corresponding to all experimental states are greater than 0.05, we considered the RACIPE model to be unassigned, indicating that the model could not be mapped to any experimental state.

Flexibility of a GRN was defined as the differences in the distribution of the assigned gene expression states of an ensemble of 10,000 RACIPE models between the unperturbed condition and any single-gene knockdown (KD) condition. The formula to compute the flexibility is

4 $${f=\frac{1}{n}\mathop{\sum }\limits_{i=1}^{n}\sqrt{\left({\left({m}_{\text{normal}}^{u}-{m}_{\text{normal}}^{{{KD}}_{i}}\right)}^{2}+{\left({m}_{\text{AML}}^{u}-{m}_{\text{AML}}^{{{KD}}_{i}}\right)}^{2}\right)},}$$

where n is the total number of TFs in the candidate GRN, ${m}_{{normal}}^{u}$ (${m}_{{AML}}^{u}$) is the proportion of the RACIPE models mapped to the normal control (or AML) experimental state under the unperturbed condition, and ${m}_{{normal}}^{{{KD}}_{i}}$ (${m}_{{AML}}^{{{KD}}_{i}}$) is the proportion of the RACIPE models mapped to the normal control (or AML) experimental state under the KD condition of the *i*th TF.

Here, GRN was ranked according to both the accuracy and flexibility. We first obtained the first ranking indices according to the accuracy (a lower rank for high accuracy) and then the second ones according to the flexibility (a lower rank for high flexibility). The combined index is defined as the sum of both ranking indices for each GRN—the lower the combined score, the higher both the accuracy and flexibility.

### Pathway annotation

We annotated the most representative biological pathways to the GRN TFs as follows. First, we obtained the differentially expressed genes (DEGs with adjusted *p* value < 0.05) between the two groups normal controls and AML patients and only retained the DEGs that were either TFs in the network or their targets found in the corresponding TF-target gene database. Second, we applied enrichr^22^ to find 12 enriched KEGG pathways from the DEGs (adjusted p-value < $1.6\times {10}^{-10}$). From these 12 pathways, we disregarded five pathways (Pathways in cancer, Epstein-Barr virus infection, Hepatitis B, Measles, and Human papillomavirus infection), because they were either too generic (Pathways in cancer) or not directly related to AML (Epstein-Barr virus infection, Hepatitis B, Measles, and Human papillomavirus infection). We selected the following seven top-ranked pathways from the enrichment analysis: Cell cycle, p53 signaling pathway, PI3K-Akt signaling pathway, JAK-STAT signaling pathway, MAPK signaling pathway, Cellular senescence, and AMPK signaling pathway. We performed Fisher’s exact test to check whether genes from each pathway overlaps with the DEGs corresponding to each TF (both the TF and their targets in the TF-target gene database) (Fig. 1b, Supplementary data 1). Finally, we annotated each TF with the pathway that has the smallest p-value provided that the *p* value ≤ 0.1. If no significant pathway with that p-value threshold was found for a TF, the TF was unassigned to a pathway. The annotated GRN was then visualized using Cytoscape^71^, as shown in Fig. 4c.

### Modeling GRN perturbations

Single and double knockdown (KD) simulations were performed on the AML GRN using RACIPE as follows. First, 10000 RACIPE models were simulated for the AML GRN to generate the gene expression profiles for the unperturbed condition. Second, for the single KD simulations for a TF in the network, we reduced the production rate of the corresponding TF by 95% for each RACIPE model and then re-simulated the model to generate the gene expression profiles for the KD condition. Third, for double KD simulations, for each RACIPE model, we reduced the production rate of both TFs by 95% and then re-simulated the model to generate the gene expression profiles for the double KD condition. We used ridge regression to map the knockdown RACIPE simulated expressions to the two groups, normal controls and AML patients. To achieve this, first, we mapped the 10,000 RACIPE models from the unperturbed simulations to normal controls and AML patients using the method described in Methods section “*Accuracy and flexibility metrics*” and used these labeled unperturbed models to train a regression model. We then used the trained regression model to map the knockdown RACIPE simulated expressions. Afterwards, we calculated the proportion of RACIPE models mapped to each group, normal control and AML patient. The effect of TF knockdown was evaluated by the change in the proportion of the models matching the two experimental groups normal controls and AML patients, compared to the simulations from the unperturbed condition.

### Survival analysis

In order to determine whether important TFs identified by our algorithm are associated with complete remission in AML, we used gene expression and clinical information for 119 primary AML patients^24^. First, a univariate Cox regression analysis was performed to evaluate the association between expression levels of genes and event-free survival of AML patients (event denotes failure to achieve complete remission). Then, we calculated a risk score for each sample which was defined as a linear combination of expression values of genes in one signature set weighted by their estimated Cox model regression coefficients. If the risk score for one sample was larger than the median risk scores, then it was classified into a high-risk group, otherwise into a low-risk group. Finally, Kaplan-Meier survival estimation and log-rank test were applied to evaluate the differences in patients’ survival time between the high-risk group and the low-risk groups^72^.

### Reporting summary

Further information on research design is available in the Nature Research Reporting Summary linked to this article.

### Supplementary information

Supplementary Information

Supplementary Data 1

Supplementary Data 2

Reporting summary

## Data Availability

Data for network modeling are available at https://github.com/lusystemsbio/AML.GRN.modeling. The optimal gene regulatory network for AML with IDH mutations is available at the Network Data Exchange portal https://www.ndexbio.org/viewer/networks/962c57d6-c5f2-11ee-8a13-005056ae23aa. The microarray gene expression data for AML patients and the ATAC-seq profiles for normal and AML samples are publicly available from the NCBI Gene Expression Omnibus under accession numbers GSE6891 and GSE74912.

## Code availability

R code for network construction, optimization, modeling and data analysis is available at https://github.com/lusystemsbio/AML.GRN.modeling.
