# Supplementary material for: Data-driven modeling of core gene regulatory network underlying leukemogenesis in IDH mutant AML
Source: NPJ Syst Biol Appl. 2024 Apr 9;10:38. doi: 10.1038/s41540-024-00366-0 (PMC11003984; doi:10.1038/s41540-024-00366-0)
Supplement: Supplementary file 1 — Supplementary Information [file 41540_2024_366_MOESM1_ESM.pdf]

Data-driven modeling of core gene regulatory network underlying leukemogenesis in IDH mutant AML

Ataur Katebi, Xiaowen Chen, Daniel Ramirez, Sheng Li, and Mingyang Lu

Supplementary Information

Supplementary Tables

Supplementary Table 1. Public datasets used in this study.

Datasets	GEO Accession Number/databases	Usage
Microarray gene expression data	GSE6891[1,2]	TF activity inference, differential expressed gene analysis, gene set enrichment, network construction
ATAC-seq data	GSE74912[3]	Context-specific TF-target gene relationships
TF-target databases	TRRUST[4], RegNet[5], and TFactS[6], TRED[7], FANTOM5[8], ChEA[9], TRANSFAC[10], JASPAER[11], ENCODE[12], and RcisTarget[13]	General databases for TF-target gene relationships

Supplementary Table 2. The choice of optimization hyperparameters.

Parameters	Values
TF binding probability (ATAC-seq)	0.05, 0.06, 0.07, 0.08, 0.09, 0.10, 0.12, 0.14, 0.16, 0.18, and 0.20 (11 values)
Number of TFs per method (NetAct, VIPER, RI)	4 to 60 at an interval of 4 (15 values)
TF activity correlation cutoff	0.0 to 0.95 at an interval of 0.05 (20 values)

Supplementary Figures

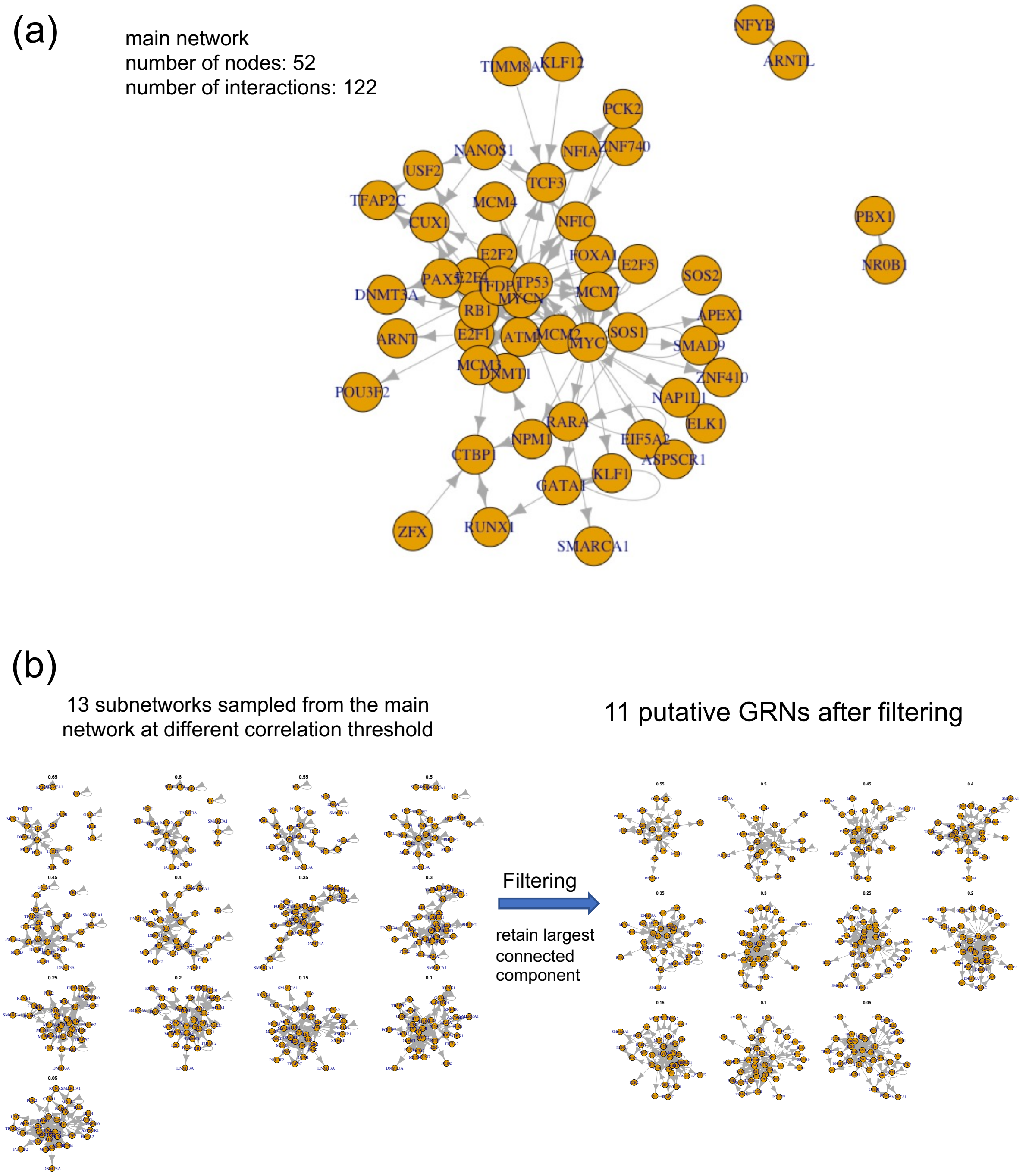

Supplementary Fig. 1. (related to Fig. 1) Inferred putative GRNs. (a) Initial network with 52 nodes and 122 interactions. **(b)** Left plot shows 13 GRNs obtained at different TF activity correlation threshold from the initial network shown on panel **a**. Right plot shows, from the 13 GRNs, the largest subnetworks, each of which have more than 80 percent of the nodes of the corresponding sampled network.

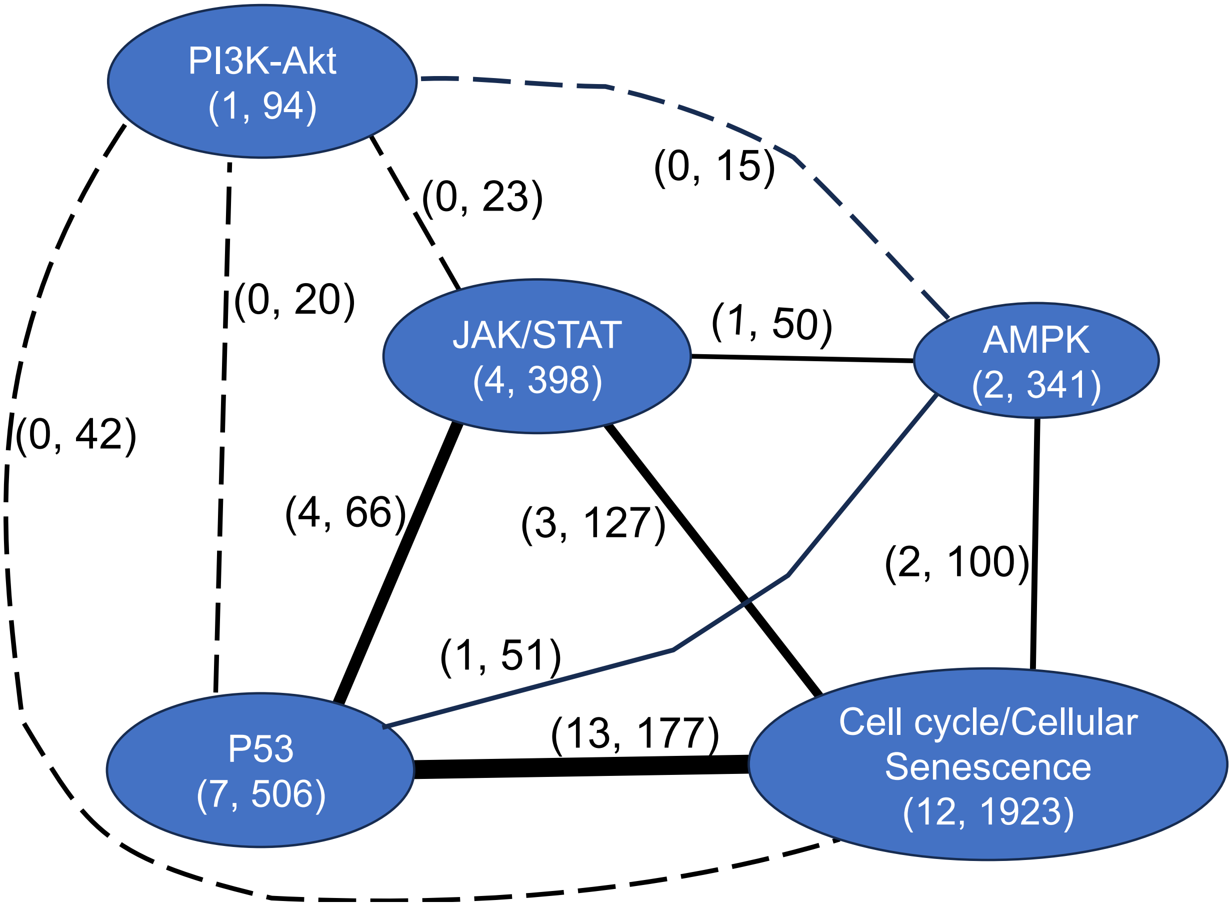

Supplementary Fig. 2. (related to Fig. 4) Coupling of biological pathways associated with the optimal GRN. Nodes represent pathways as labeled. The two numbers within each node represent the number of transcription factors (TFs) corresponding to that pathway and the total number of genes targeted by those TFs, respectively. The two numbers on each edge label represent number of links between the two TF groups and the number of common target genes targeted by the TF groups. Thicker edges indicate comparatively more TF links between the two groups. Dotted edge indicates no links found between the two TF groups. Target gene names by each TF in the optimal GRN are listed in **Supplementary Table 4**.

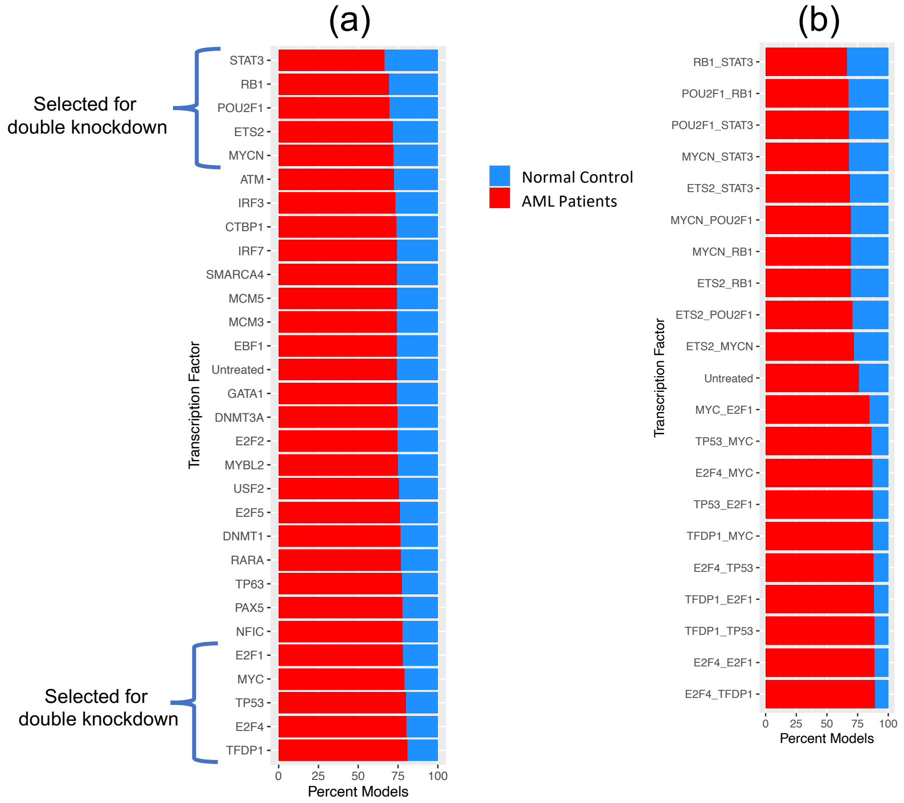

Supplementary Fig. 3. (related to Fig. 6a) Gene perturbation simulations on the optimal GRN. RACIPE model proportions (ordered) for (a) single knockdown and (b) double knockdown simulations. Blue proportions represent RACIPE models mapped to the samples of the normal control. The red proportions represent RACIPE models mapped to the samples of the AML patients.

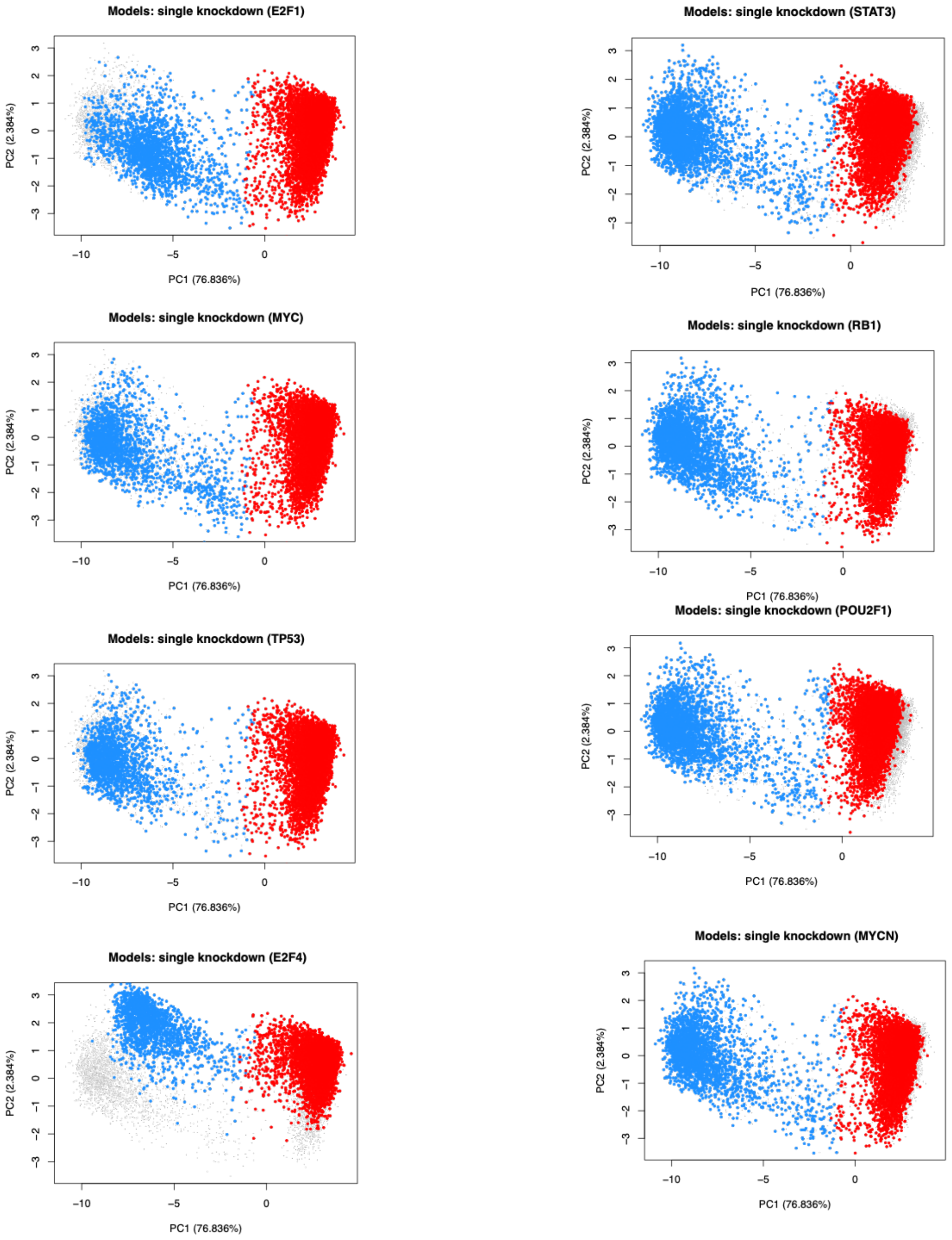

Supplementary Fig. 4. (related to Fig. 6c) Examples of changes in gene expression profiles upon single knockdown perturbations. Left: Perturbations that increase AML proportions. Right: Perturbations that decrease AML proportions.

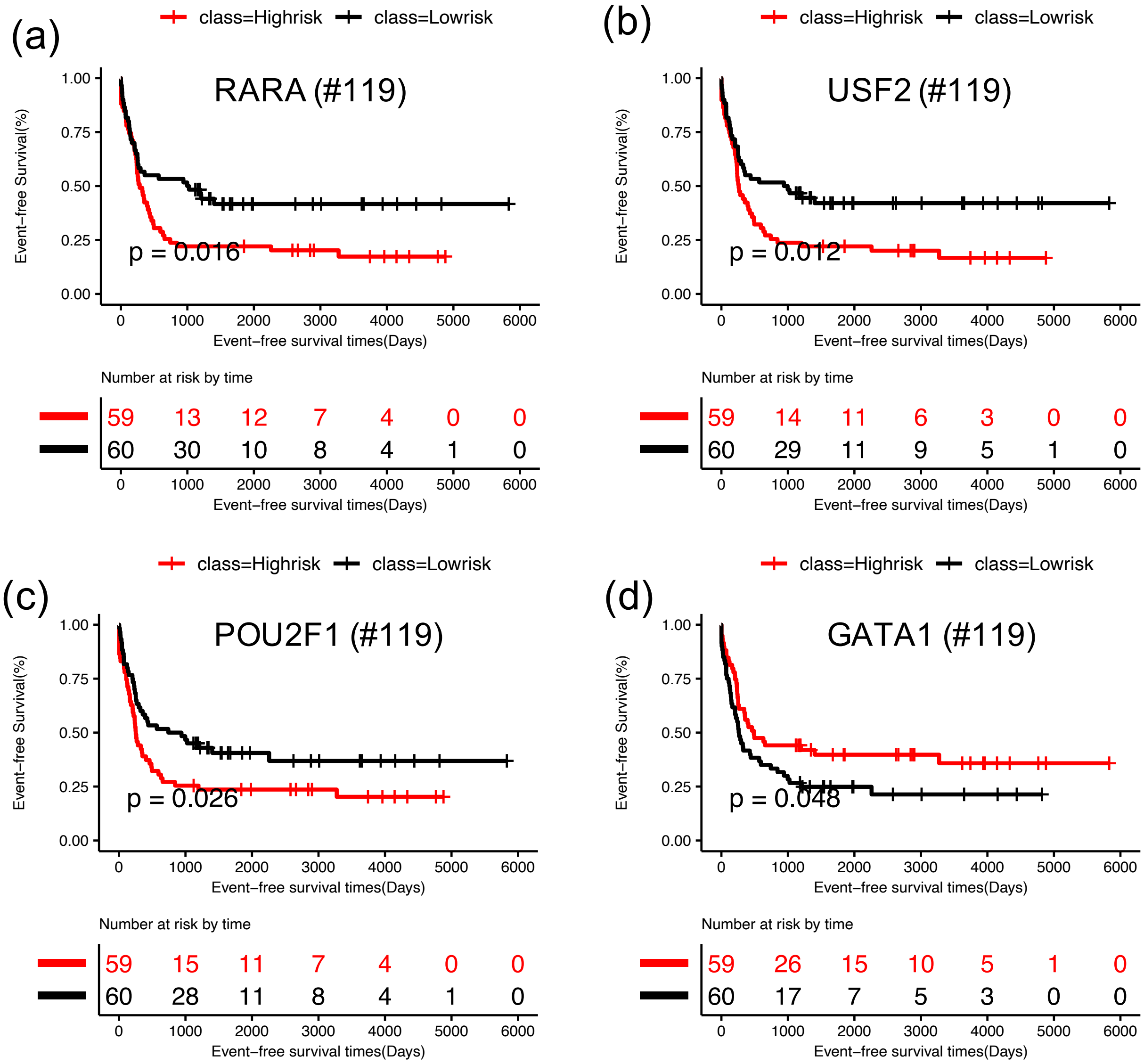

Supplementary Fig. 5. (related to Fig. 7) Additional results for patient survival analysis. Kaplan-Meier curves for event free survival for individual TFs on the AML GRN using all 119 AML patients with p-value <= 0.05. (a) RARA, (b) USF2, (c) POU2F1, and (d) GATA1.

Supplementary References:

1. Glass, J. L. *et al.* Epigenetic identity in AML depends on disruption of nonpromoter regulatory elements and Is affected by antagonistic effects of mutations in epigenetic modifiers. *Cancer Discovery* **7**, 868–883 (2017).
2. Verhaak, R. G. W. *et al.* Prediction of molecular subtypes in acute myeloid leukemia based on gene expression profiling. *1* **94**, 131–134 (2009).
3. Corces, M. R. *et al.* Lineage-specific and single-cell chromatin accessibility charts human hematopoiesis and leukemia evolution. *Nature Genetics* **48**, 1193–1203 (2016).
4. Han, H. *et al.* TRRUST: a reference database of human transcriptional regulatory interactions. *Scientific Reports* **5**, 11432 (2015).
5. Chi, S.-M. *et al.* REGNET: mining context-specific human transcription networks using composite genomic information. *BMC Genomics* **15**, 450 (2014).
6. Essaghir, A. *et al.* Transcription factor regulation can be accurately predicted from the presence of target gene signatures in microarray gene expression data. *Nucleic Acids Research* **38**, e120–e120 (2010).
7. Jiang, C., Xuan, Z., Zhao, F. & Zhang, M. Q. TRED: a transcriptional regulatory element database, new entries and other development. *Nucleic Acids Research* **35**, D137–D140 (2007).
8. Abugessaisa, I. *et al.* FANTOM5 transcriptome catalog of cellular states based on Semantic MediaWiki. *Database (Oxford)* **2016**, baw105 (2016).
9. Lachmann, A. *et al.* ChEA: transcription factor regulation inferred from integrating genome-wide ChIP-X experiments. *Bioinformatics* **26**, 2438–2444 (2010).
10. Wingender, E., Dietze, P., Karas, H. & Knüppel, R. TRANSFAC: A Database on Transcription Factors and Their DNA Binding Sites. *Nucleic Acids Research* **24**, 238–241 (1996).
11. Sandelin, A., Alkema, W., Engström, P., Wasserman, W. W. & Lenhard, B. JASPAR: an open-access database for eukaryotic transcription factor binding profiles. *Nucleic Acids Research* **32**, D91–D94 (2004).

12. Luo, Y. *et al.* New developments on the Encyclopedia of DNA Elements (ENCODE) data portal. *Nucleic Acids Res* **48**, D882–D889 (2020).
13. Aibar, S. *et al.* SCENIC: single-cell regulatory network inference and clustering. *Nat Methods* **14**, 1083–1086 (2017).
